# Pelvic radiography in ATLS algorithms: A diminishing role?

**DOI:** 10.1186/1749-7922-3-11

**Published:** 2008-03-04

**Authors:** Matthias P Hilty, Isabelle Behrendt, Lorin M Benneker, Luca Martinolli, Christoforos Stoupis, Donald J Buggy, Heinz Zimmermann, Aristomenis K Exadaktylos

**Affiliations:** 1 Department of Emergency Medicine, University Hospital of Berne, Switzerland; 2 Department of Orthopedic Surgery, University Hospital of Berne, Switzerland; 3 Institute of Diagnostic Radiology, University Hospital of Berne, Switzerland; 4 Department of Anaesthesia, Mater Misericordiae University Hospital, Dublin, Ireland

## Abstract

**Background:**

Pelvic x-ray is a routine part of the primary survey of polytraumatized patients according to Advanced Trauma Life Support (ATLS) guidelines. However, pelvic CT is the gold standard imaging technique in the diagnosis of pelvic fractures. This study was conducted to confirm the safety of a modified ATLS algorithm omitting pelvic x-ray in hemodynamically stable polytraumatized patients with clinically stable pelvis in favour of later pelvic examination by CT scan.

**Methods:**

We conducted a retrospective analysis of all polytraumatized patients in our emergency room between 01.07.2004 and 31.01.2006. Inclusion criteria were blunt abdominal trauma, initial hemodynamic stability and a stable pelvis on clinical examination. We excluded patients requiring immediate intervention because of hemodynamic instability.

**Results:**

We reviewed the records of n = 452 polytraumatized patients, of which n = 91 fulfilled inclusion criteria (56% male, mean age = 45 years). The mechanism of trauma included 43% road traffic accidents, 47% falls. In 68/91 (75%) patients, both a pelvic x-ray and a CT examination were performed; the remainder had only pelvic CT. In 6/68 (9%) patients, pelvic fracture was diagnosed by pelvic x-ray. None of these 6 patients was found having a false positive pelvic x-ray, i.e. there was no fracture on pelvic CT scan. In 3/68 (4%) cases a fracture was missed in the pelvic x-ray, but confirmed on CT (false negative on x-ray). None of the diagnosed fractures needed an immediate therapeutic intervention. 5 (56%) were classified type A fractures, and another 4 (44%) B 2.1 in computed tomography (AO classification). One A 2.1 fracture was found in a clinically stable patient who only received CT scan (1/23).

**Conclusion:**

While pelvic x-ray is an integral part of ATLS assessment, this retrospective study suggests that in hemodynamically stable patients with clinically stable pevis, its sensitivity is only 67% and it may safely be omitted in favor of a pelvic CT examination if such is planned in adjunct assessment and available. The results support the safety and utility of our modified ATLS algorithm. A randomized controlled trial using the algorithm can safely be conducted to confirm the results.

## Background

Blunt abdominal trauma is a leading cause of morbidity and mortality among all age groups [1,2]. Identification of serious intraabdominal pathology is often challenging and injury to abdominal and pelvic structures can be classified into two primary mechanisms: compression forces and deceleration forces. Both frequently cause intraabdominal injuries and pelvic fractures [3]. Because of the risk of severe bleeding which can lead to hypovolaemic shock, coagulopathy and death, a high index of suspicion for pelvic fracture must be maintained.

In the 7^th ^Edition of the ATLS Guidelines (2004), three x-rays are recommended as part of the primary survey: cervical spine, thorax and pelvis. Even though the guidelines leave some room for interpretation, pelvic x-ray is generally thought to be mandatory in polytraumatized patients although it may not give a definitive diagnosis of pelvic fractures [4,5].

Because intraabdominal lesions are hard to exclude without a CT examination, a CT scan of the entire abdomen and pelvis is often performed in the secondary survey [6]. It has already been discussed to omit either the clinical examination of the pelvis in the primary survey or the pelvic x-ray since the patients will undergo CT survey in adult and paediatric trauma patients [7-9], since it is vital for the survival of polytraumatized patients to follow a fast and structured approach in diagnosis and therapy.

**Figure 1:**
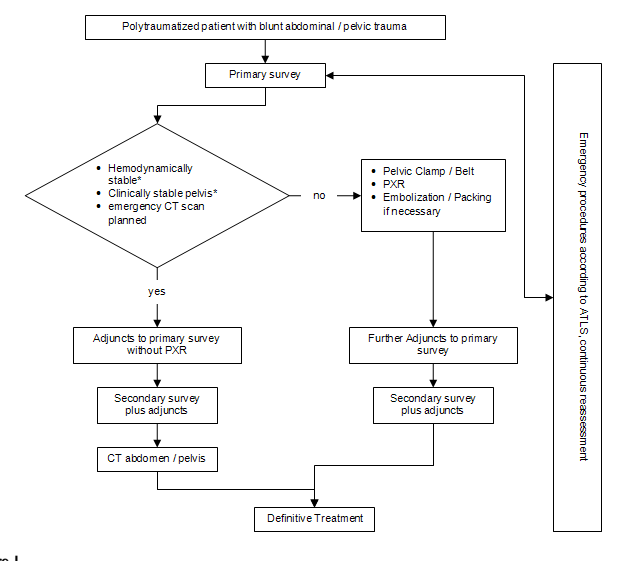
**Management algorithm focusing on pelvic fractures**. PXR: Pelvic x-ray; * see definition under "Methods"

Therefore, there is a need for further evidence-based guidelines in this area. We have devised a modified ATLS algorithm omitting pelvic x-ray in hemodynamically stable polytraumatized patients with clinically stable pelvis in favour of later pelvic examination by CT scan (see Figure 1). The objective of this study was to confirm the safety of the algorithm by comparing the incidence of pelvic fractures diagnosed by pelvic x-ray, using the pelvic CT as gold standard, in hemodynamically stable polytraumatized patients who sustained blunt abdominal trauma.

## Methods

A retrospective chart analysis of all multiple injured (ISS ≥ 16) patients admitted to our emergency room between 01.07.2004 and 31.01.2006 was conducted. Inclusion criteria were blunt abdominal/pelvic trauma, initial hemodynamic stability, and a stable pelvis on clinical examination. Information on age, sex, mechanism and severity of trauma, initial condition of the patient and treatment during the first hour, x-ray findings (if performed), CT findings, other diagnoses, definitive treatment and outcome were collected and compared. The pelvic fractures were classified according to Tiles AO fracture classification modified by Isler and Ganz [10,11].

Our unit is a level I emergency medicine facility serving env. 1.5 million people and seeing about 30.000 trauma patients per year. All senior attending physicians are ATLS certified and the protocols are implemented. Clinical pelvic examination is always performed by a senior resident or the attending consultant emergency physician who is the trauma team leader. CT scans were performed using a high-end device yielding thin sections and coronal reconstructions. CT interpretation was done by experienced radiologists.

We defined hemodynamic stability as hemorrhagic shock not worse than Class 2, i.e. patients were normotensive, had elevated or normal pulse rate, respiratory rate <30/min, normal or decreased pulse pressure (art. pulse amplitude), and had a rapid response to the initial fluid therapy of 2 L crystalloid. The finding of clinically stable pelvis in our definition includes that the pelvis does not give way during the application of vertical, horizontal or lateral shearing forces and that such compression is not painful, that there is no perineal hematoma, no open wounds in the pelvic region and that no blood is visible exiting the urethral meatus.

## Results

Over the evaluated period of 18 months our trauma team treated 452 polytraumatized patients with blunt abdominal trauma. 91 of them fulfilled the inclusion criteria. The mean patient age was 45 years, and 44% were women. The mechanism of trauma was road traffic accident in 47% and fall in 43%. Mean Injury Severity Score (ISS) was 23, with a range of 17 to 50. 78 patients (86%) initially had hemorrhagic shock class 0 or 1, 13 patients (14%) class 2. All responded rapidly to initial fluid resuscitation (2 L of warmed ringer's lactate).

68 (75%) of the 91 overall patients received both a pelvic x-ray and a CT examination (group I). In 6 patients (9%) of group I, a pelvic fracture was diagnosed through PXR; in all of those cases the finding was confirmed in the CT examination – no false positive PXR were observed. In 3 patients of group I (4%) a pelvic fracture was missed in the pelvic x-ray. Two of these patients with missed fractures were initially x-rayed and assessed in peripheral hospitals and transferred to our unit for CT examination. One fracture was classified A 2.1 (undisplaced fractures of ramus superior of os pubis); the second was a A 1.2 type (minimal displaced fracture of the iliac wing). Both were easily detected on retrospective evaluation by an experienced trauma surgeon. The third missed fracture was classified A 2.2 (unilateral minimal compression fracture of the massa lateralis of the sacrum) and could not be detected even on retrospective evaluation of pelvic x-ray. Overall in group I one A 1.2, three A 2.1, one A 2.2, and four B 2.1 were diagnosed by CT examination. Specificity of pelvic x-ray in relation to CT was found to be 100% and sensitivity 67% (see Table 1).

**Table 1:** Sensitivity and specifity of PXR in relation to CT

	*Fx pos (CT)*	*Fx neg (CT)*
*PXR pos*	6	0
*PXR neg*	3	59

	Sensitivity: 0.67	Specifity: 1

PXR: Pelvic x-ray

Group II consists of 23 patients (25%) who directly received a CT examination without pelvic x-ray. In 1 case (4%), a pelvic fracture, classified A 2.1, was found.

70 (77%) of the patients in group I and II presented with additional head/CNS injury, 18 (20%) with additional spine injury, 31 (34%) with additional thoracic injury and 7 (8%) with additional intraabdominal injury.

None of the patients in group II suffered any complications because of delayed diagnosis of pelvic fractures, and none of the patients of group I or II required an immediate operative or radiological intervention because of pelvic injury, such as pelvic clamp, packing or intra-arterial coil embolization. 2 patients in group I and none in group II died of complications in other organ systems. The patient diagnosed with A 2.2 pelvic fracture needed iliosacral screw fixation ten days after initial treatment.

## Discussion and Conclusion

This retrospective data suggests that pelvic x-ray can safely be avoided in hemodynamically stable patients with clinically stable pelvis. Firstly, patients who received both a pelvic x-ray and a CT scan and those who received a CT scan only, had identical outcome. Secondly, none of the patients who received a subsequent CT scan only, had any acute complications. One case with an A 2.1 pelvic fracture, which was clinically stable and not bleeding, was missed in the clinical examination during the primary survey and might have been detected earlier using conventional radiography after the primary survey.

Even though conventional pelvic x-ray in Emergency Departments was shown to have a specifity of 100% compared with CT, 33% of all pelvic fractures are missed by pelvic x-ray. Our present study suggests that pelvic x-ray may be of limited value in this context. It could be studied if the missed fractures are of minor importance compared to the ones detected. Patients who suffer pelvic fractures are usually the victims of trauma and often have other trauma-related injuries. Up to 52% of emergency department patients with pelvic fractures develop shock due to hemorrhage and despite the escalating role of computed tomography in trauma, conventional radiography remains important in the acute management of trauma patients [7]. Current ATLS guidelines recommend that only chest, pelvis and lateral cervical spine are x-rayed immediately. Inherent delays, relatively high radiation dose and practical difficulties in acquiring radiographs in the multi-injured patient can adversely affect management, bearing in mind the critical importance of physician access to badly injured patients during the "golden hour".

Conventional pelvic radiography has a radiation of up to 20% of that of a spiral CT of the region [12], so radiation reduction by omission of conventional studies is significant. The potential cost reduction of omitting pelvic x-rays routinely is also significant.

Abdominal injuries influence the diagnostic procedures in blunt trauma. Focused assessment with sonography in trauma (FAST), introduced a decade ago, is non invasive and has high sensitivity but low specificity for the detection of free fluid and visceral damage [6]. This may have an impact on treatment and outcome in trauma patients. CT remains the diagnostic study of choice at most institutions even in the evaluation of hemodynamically stable, blunt abdominal trauma. It is highly specific and sensitive in the detection and definition of the extent of most intra-abdominal and also pelvic injuries but requires the patient to be hemodynamically stable according to ATLS principles.

A trend towards the avoidance of multiple radiographic studies and to early utilisation of CT instead is occurring [13,14], partly because of the ability of CT to define the extent of injury better than any other immediately available radiographic tool and because CT scanners have become much faster and integrated into many modern emergency rooms. In our population group, all potentially hemorrhagic pelvic fractures (AO classification B1 and C) could be ruled out by clinical examination and the other inclusion criteria. The remaining fractures have little potential for acutely life-threatening complications and resulted from direct anterior (A2.1) or lateral (B2.1) compression instead of rotational forces in the vertical plane. Recent studies with similar hypothesis and different patient population support our results [7-9,15].

In conclusion, this retrospective study suggests that pelvic x-ray has limited value for detecting pelvic fractures compared with CT scanning. The clinical examination of the abdomen and pelvis remains a key factor in the decision making regarding further radiological examinations. Hemodynamically stable patients who undergo CT scanning after immediate resuscitation and have a stable pelvis on clinical examination seem not to benefit from a routine pelvic x-ray. Pelvic x-ray remains important in the management of hemodynamically unstable patients and patients with suspect clinical examination of the pelvis. The results support the safety and utility of our modified ATLS algorithm described above (Figure 1). A randomized controlled trial using the algorithm can safely be conducted to confirm the results.

## References

[B1] Hammel J, Legome E (2006). Pelvic fracture. J Emerg Med.

[B2] Muller EJ, Siebenrock K, Ekkernkamp A, Ganz R, Muhr G (1999). Ipsilateral fractures of the pelvis and the femur – floating hip? A retrospective analysis of 42 cases. Arch Orthop Trauma Surg.

[B3] Demetriades D, Karaiskakis M, Toutouzas K, Alo K, Velmahos G, Chan L (2002). Pelvic fractures: epidemiology and predictors of associated abdominal injuries and outcomes. J Am Coll Surg.

[B4] Velmahos GC, Jindal A, Chan LS, Murray JA, Vassiliu P, Berne TV, Asensio J, Demetriades D (2001). "Insignificant" mechanism of injury: not to be taken lightly. J Am Coll Surg.

[B5] Young JW, Burgess AR, Brumback RJ, Poka A (1986). Pelvic fractures: value of plain radiography in early assessment and management. Radiology.

[B6] Miller MT, Pasquale MD, Bromberg WJ, Wasser TE, Cox J (2003). Not so FAST. J Trauma.

[B7] Guillamondegui OD, Pryor JP, Gracias VH, Gupta R, Reilly PM, Schwab CW (2002). Pelvic radiography in blunt trauma resuscitation: a diminishing role. J Trauma.

[B8] Vo NJ, Gash J, Browning J, Hutson RK (2004). Pelvic imaging in the stable trauma patient: is the AP pelvic radiograph necessary when abdominopelvic CT shows no acute injury?. Emerg Radiol.

[B9] Guillamondegui OD, Mahboubi S, Stafford PW, Nance ML (2003). The utility of the pelvic radiograph in the assessment of pediatric pelvic fractures. J Trauma.

[B10] Isler B, Ganz R (1996). Classification of pelvic ring injuries. Injury.

[B11] Tile M (1995). Fractures of the pelvis and Acetabulum: principles of management.

[B12] Jurik AG, Jensen LC, Hansen J (1996). Total effective radiation dose from spiral CT and conventional radiography of the pelvis with regard to fracture classification. Acta Radiol.

[B13] Rommens P, Wissing H, Serdarevic M (1987). [Significance of computerized tomography in the diagnosis and therapy of fractures of the posterior pelvic ring and hip joint]. Unfallchirurgie.

[B14] Dunn EL, Berry PH, Connally JD (1983). Computed tomography of the pelvis in patients with multiple injuries. J Trauma.

[B15] Obaid AK, Barleben A, Porral D, Lush S, Cinat M (2006). Utility of plain film pelvic radiographs in blunt trauma patients in the emergency department. Am Surg.

